# New role of ID3 in melanoma adaptive drug-resistance

**DOI:** 10.18632/oncotarget.22698

**Published:** 2017-11-27

**Authors:** Sachindra, Lionel Larribère, Daniel Novak, Huizi Wu, Laura Hüser, Karol Granados, Elias Orouji, Jochen Utikal

**Affiliations:** ^1^ Skin Cancer Unit, German Cancer Research Center (DKFZ), Heidelberg, Germany; ^2^ Department of Dermatology, Venereology and Allergology, University Medical Center Mannheim, Ruprecht-Karls University of Heidelberg, Mannheim, Germany; ^3^ Department of Pharmacy, Xiangya Hospital, Central South University, Changsha, China; ^4^ Department of Genomic Medicine, University of Texas MD Anderson Cancer Center, Houston, TX, USA

**Keywords:** melanoma, ID3, drug-resistance, targeted therapy, BRAF

## Abstract

Adaptive resistance to targeted therapy such as BRAF inhibitors represents in melanoma a major drawback to this otherwise powerful treatment. Some of the underlying molecular mechanisms have recently been described: hyperactivation of the BRAF-MAPK pathway, of the AKT pathway, of the TGFβ/EGFR/PDGFRB pathway, or the low MITF/AXL ratio. Nevertheless, the phenomenon of early resistance is still not clearly understood. In this report, we show that knockdown of neural crest-associated gene *ID3* increases the melanoma sensitivity to vemurafenib short-term treatment. In addition, we observe an *ID3*-mediated regulation of cell migration and of the expression of resistance-associated genes such as *SOX10* and *MITF*. In sum, these data suggest ID3 as a new key actor of melanoma adaptive resistance to vemurafenib and as a potential drug target.

## INTRODUCTION

Most skin cancer related deaths are attributed to melanoma. It is responsible for more than 75% of death while it represents 4% of all skin cancers only [1]. The genetic causes of melanoma invasion have been intensively studied and in particular the hyperactivation of the MAPK pathway has been well characterized [2]. Since the discovery of *BRAF* mutations in melanoma patients (50-60%), numerous small molecule inhibitors have been tested on melanoma [3]. BRAF inhibitors such as vemurafenib or dabrafenib were approved by FDA and EMA to treat advanced melanoma patients with ^V600^*BRAF* mutations [4]. Vemurafenib has led to an increase in the rates of progression-free (PFS: 5.3 months) and overall survival (OS: 34% at 6 months) in stage III clinical trials compared with conventional chemotherapy in patients with *BRAF*-mutated metastatic melanoma [5]. However, resistance to BRAF inhibitors, based on the Response Evaluation Criteria In Solid Tumors (RECIST; [6]), could occur within 6 to 7 months and therefore combinations with MEK inhibitors such as trametinib or cobimetinib were later approved by FDA and EMA [7–11]. The 3-year PFS in the combination group (dabrafenib plus trametinib) was 22% and only 12% in the monotherapy group. The 3-year OS with combination therapy was 44% versus 32% respectively. However, most of the patients from the combination group develop progressing disease. Besides, up to 20% melanoma patients with *BRAF* mutations do not respond to vemurafenib treatment at all, probably due to intrinsic resistance mechanisms such as amplification of tumor promoter genes or loss of tumor suppressor genes [12].

Major investigations are currently ongoing to understand the resistance mechanisms acquired during BRAF inhibitor monotherapy and BRAF/ MEK inhibitor combination therapy. Several models such as primary human melanoma xenograft models or vemurafenib resistant melanoma cell lines have been established and extensively studied [13]. While acquired resistance is established in a tumor after drastic shrinkage followed by a rapid regrowth, the term adaptive resistance relates to the mechanisms that are activated in a short time after drug administration to the tumor and which would represent an initial step towards acquired resistance [14]. For example, downregulation of negative regulators of the RAS-RAF-MAPK pathway such as DUSP or SPRY was described during adaptive resistance in melanoma [15]. Moreover, activation of AKT pathway via an increase of PDGFRb or ERBB3 was also shown to participate to a rapid response to RAF inhibitors [16]. On the other hand, activation of cAMP signalling or low MITF/AXL expression ratio are involved in melanoma acquired resistance to vemurafenib [17, 18].

Inhibitor of differentiation protein 3 (*ID3)* is part of the *ID* gene family of helix-loop-helix (HLH) transcription factors which are considered as negative regulators of transcription [19]. *ID3* is involved in cell cycle progression and survival of neural crest progenitors [20]. This gene also plays a key role in various cancer types including melanoma [21]. In this report, we identify *ID3* as a new molecule involved in melanoma adaptive resistance to vemurafenib and in the regulation of melanoma migration.

## RESULTS

### ID3 expression regulates melanoma adaptive resistance to vemurafenib

**Figure 1:**
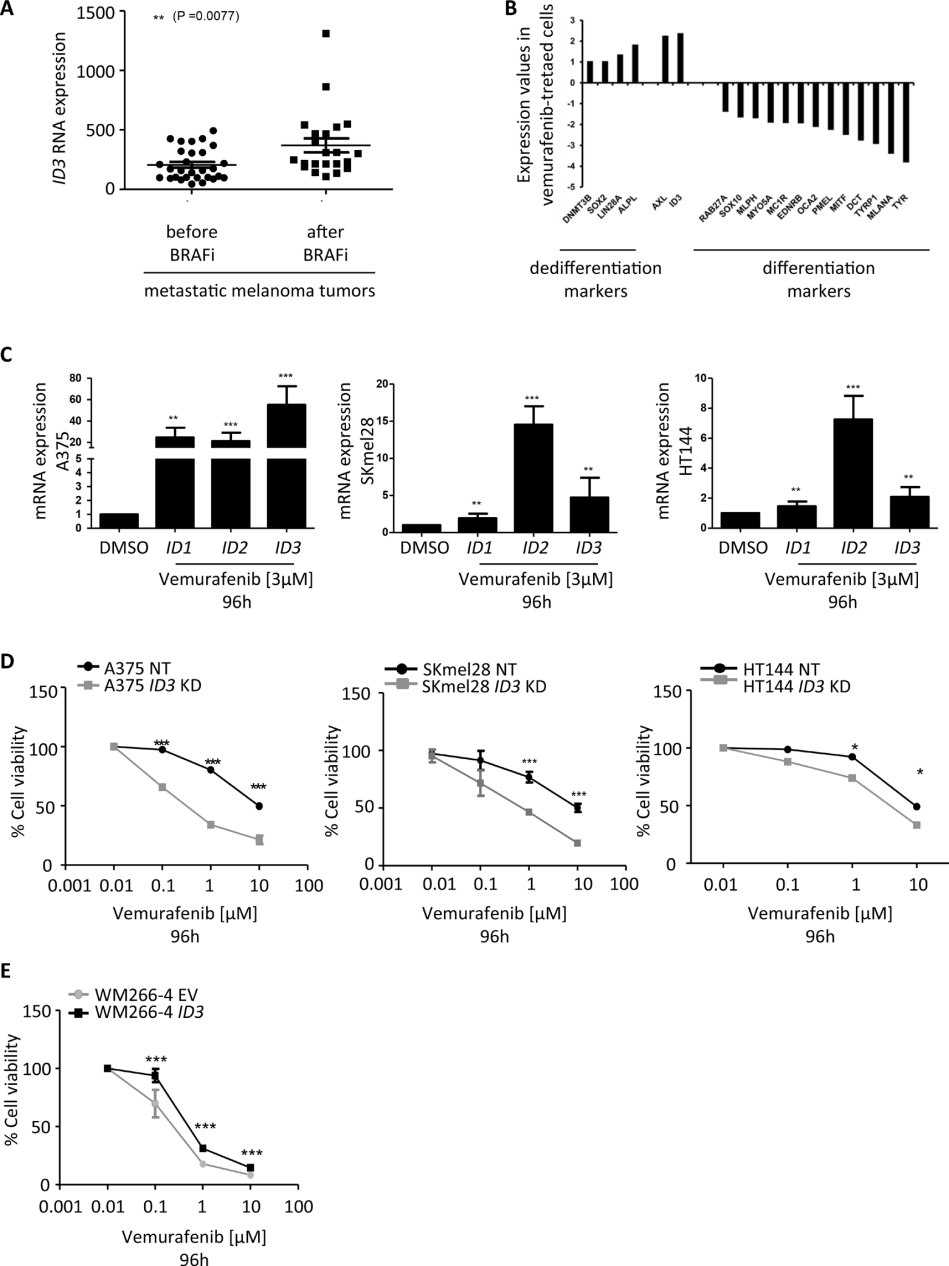
*ID3* expression regulates melanoma adaptive resistance to vemurafenib (**A**) *ID3* RNA expression analysed in tumor samples derived from progressing melanoma patients before and after BRAF inhibitor treatment (GSE50509). **P < 0.0077. (**B**) Gene expression values of differentiation and dedifferentiation markers, as well as *AXL* and *ID3* shown as a fold change of the dedifferentiated cells samples (average of vemurafenib-treated cells and D1NC) compared to the differentiated cells samples (average of DMSO-treated cells and NHM). (**C**) *ID1, ID2* and *ID3* mRNA expression in human melanoma cell lines (A375, SKmel28, HT144) treated with 3 μM vemurafenib for 96 h. rRNA *18S* was used as an endogenous expression control and DMSO treated cells were used as reference sample. (**D**) Graph represents the effect of vemurafenib treatment (0.01-10 μM) after 96 hours on the viability of *ID3* knockdown cell lines (*ID3* KD) or cell lines transduced with a non-targeting shRNA (NT), assessed by Alamar blue staining (A375, SKmel28 and HT144). (**E**) Graph represents the effect of vemurafenib treatment (0.01-10 μM) after 96 hours on the viability of *ID3* overexpressing cell line (WM266-4 *ID3*) or the cell line transduced with an empty vector (WM266-4 EV). C. to E.: Data are shown as mean ± SD of biological triplicates. ^*^P < 0.05, ^**^P < 0.01, ^***^P < 0.001.

A recently published study analysed the transcriptome profile of *BRAF*-mutated melanoma tumors derived from 21 patients either at the beginning of their treatment with BRAF inhibitors or at the time of disease progression. The resistance mechanisms developed by the tumors in the course of the treatment were investigated and most were involved in MAPK pathway activation [22]. Among the main regulated genes, we found *ID3* significantly upregulated in the resistant tumors compared to before treatment (*p* = 0.0077) and this upregulation (fold change > 2) was observed in 38% of the patients (Figure 1A). On the same note, we observed in our laboratory, a 2-fold upregulation of *ID3* expression in vemurafenib-treated melanoma cell lines compared to DMSO treatment. In this experiment, we compared the gene expression profile of several samples: *BRAF*-mutated melanoma cell lines after a short-term vemurafenib treatment (A375, SKmel28, WM266-4), DMSO-treated cell lines, differentiated cells such as normal human melanocytes (NHM) and less differentiated cells such as pluripotent stem cell-derived neural crest cells (D1NC) generated in our laboratory [23, 24]. An unsupervised hierarchical clustering showed that vemurafenib-treated cell lines’ expression profile had closer similarities to the profile of D1NC (this cluster was named “dedifferentiated cells”) than to the profile of NHM, which grouped together with DMSO-treated control cell lines (this cluster was named “differentiated cells”) (Supplementary Figure 1A). In addition to *ID3* upregulation, we also observed in this analysis an upregulation (log2-fold change ranging from 1 to 2) of pluripotency markers (*SOX2*, *LIN28*, *DNMT3B* and *ALPL*) and of *AXL* in the vemurafenib-treated cell lines compared to DMSO-treated control cells. Conversely, we observed a downregulation (log2-fold change ranging from -1.4 to -3.8) of differentiation markers (*MITF, DCT, TYRP1, TYR, MC1R, MLANA, SOX10, RAB27A, MLPH, MYO5A, EDNRB, OCA2, PMEL*) (Figure 1B). Moreover, a deeper analysis (Ingenuity Pathway Analysis) of the regulated genes between vemurafenib-treated melanoma lines and the lines treated with DMSO, revealed many genes involved in the cell cycle regulation (Cyclins and checkpoints regulators) or DNA damage response (ATM, p53) which could be expected after a treatment with an inhibitor of the MAPK pathway such as vemurafenib. Interestingly, Glycolysis and Wnt/βcatenin signaling pathways were also found predominantly regulated by vemurafenib treatment (Supplementary Figure 1B). Thus, these preliminary data suggested that vemurafenib treatment leads to dedifferentiation of melanoma cells and to an upregulation of *ID3* expression.

To confirm the potential role of *ID3* in the response of melanoma cells to drug treatment, we treated several melanoma cell lines with vemurafenib or in combination with trametinib. The design of this experiment was the following: four *BRAF*-mutated melanoma cell lines (A375, SKmel28, HT144 and WM266-4) and one *BRAF*-WT cell line (SKmel23) were treated with increasing doses of vemurafenib for 96h (Supplementary Figure 2A). As expected, the four *BRAF*-mutated cell lines were more sensitive to the treatment than the *BRAF*-WT cell line, used as a control. In particular, the treatment with 3 μM vemurafenib for 96h, led to 20% surviving cell for WM266-4, to 25% for A375, to 18% for SKmel28 and 44% for HT144, in comparison to SKmel23 cell line in which 82% cells survived (Supplementary Figure 2B and Supplementary Table 1).

Based on these results, we used a treatment of 3 μM vemurafenib during 96h for all the following experiments (or a combination of vemurafenib (3 μM) and trametinib (3 μM) for 96 h). We first confirmed by qPCR that not only the expression of *ID3*, but also *ID1* and *ID2* significantly increased in A375, SKmel28, and HT144, after vemurafenib or combination treatment (Figure 1C and Supplementary Figure 3A).

Next, we generated three *ID3* knockdown cell lines (A375, SKmel28, HT144) and one *ID3* overexpressing cell line (WM266-4). We verified by western blot or qPCR either silencing or overexpression of *ID3* in all genetically modified cell lines. Indeed, ID3 expression was greatly impaired in A375 and HT144 cell lines and it was silenced more than 50% in SKmel28 (Supplementary Figure 4A). Similarly, ID3 was highly overexpressed in WM266-4 cell line (Supplementary Figure 4B). We then tested these cell lines’ viability in a vemurafenib dose response assay (0.01–10 μM) after 96 h. The results showed an increased sensitivity to the drug treatment for all *ID3* knockdown cell lines compared to the control cell lines (A375, SKmel28 and HT144) (Figure 1D). Conversely, the results showed that *ID3* overexpressing cell line (WM266-4 *ID3*) became significantly more resistant to the drug treatment than the control cell line (WM266-4 EV) (Figure 1E). Of note, the combination treatment on WM266-4 *ID3* cell line also led to a significant increase of resistance compared to the control cell line WM266-4 EV (Supplementary Figure 3B).

Together, these data show that vemurafenib treatment or combination treatment with trametinib upregulate *ID3* expression and that, changes in *ID3* expression level can regulate early resistance of melanoma cells, suggesting a role for ID3 in adaptive resistance to these drugs.

### *ID3* regulates cell migration but not proliferation or cell cycle states and it also regulates *SOX10*/ *MITF* expression

**Figure 2:**
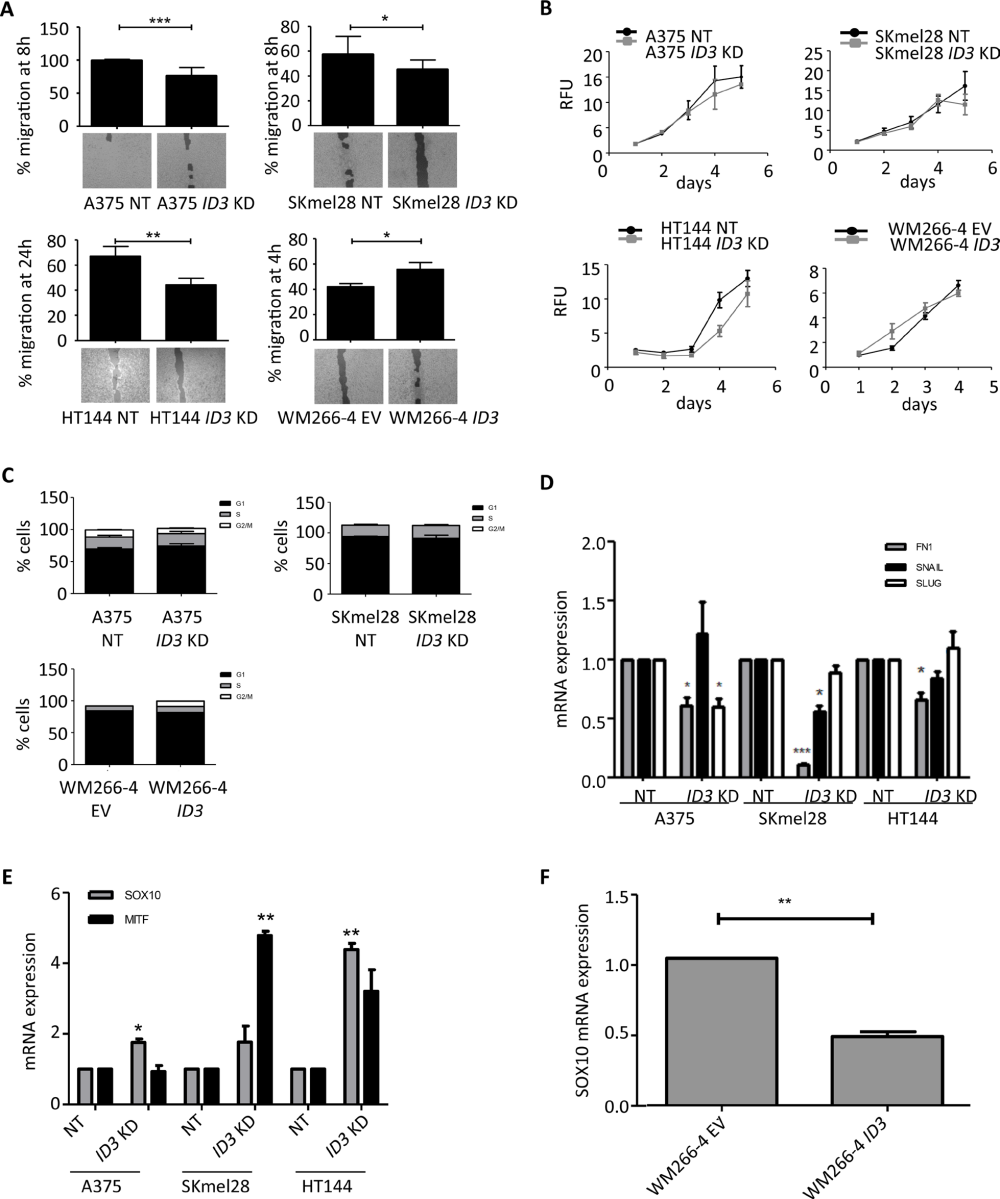
ID3 regulates cell migration but not cell proliferation or cell cycle states and it regulates SOX10/ MITF expression (**A**) Migration rate of *ID3*-engineered cell lines (A375, SKmel28, HT144 and WM266-4) was measured as a percentage of the gap closure at the indicated time points. Bottom pictures show representative images for each condition. (**B**) Cell proliferation of *ID3*-engineered cell lines (A375, SKmel28, HT144 and WM266-4) was measured by Alamar blue staining everyday up to 5 days. Relative Fluorescence Units (RFU) are represented in 10^3^ values. (**C**) Analysis of cell cycle stages by flow cytometry after PI staining of *ID3*-engineered cell lines (A375, SKmel28 and WM266-4). (**D**) Quantitative real-time PCR analysis of mesenchymal markers *FN1*, *SNAIL* and *SLUG* in *ID3* knockdown cell lines (A375, SKmel28 and HT144). (**E**) Quantitative real-time PCR analysis of *MITF* and *SOX10* expression in *ID3* knockdown melanoma cell lines (A375, SKmel28 and HT144). (**F**) Quantitative real-time PCR analysis of *SOX10* in *ID3* overexpressing WM266-4 cell line. A. – E.: Data represent mean ± SD of biological triplicates. *P < 0.05, **P < 0.01, ***P < 0.001.

To acquire more insight into the mechanism of *ID3* function in melanoma progression, we further characterized *ID3* knockdown and *ID3* overexpressing cell lines with respect to cell proliferation, cell migration and cell cycle states. Indeed, by using a scratch-like assay, we observed a delay in the migration rate of all three *ID3* knockdown cell lines (A375, SKmel28 and HT144) compared to the control cell lines, ranging from 10% to 25% reduction. Conversely, the *ID3* overexpressing cell line (WM266-4 *ID3*) migrated significantly faster than the control line (WM266-4 EV), enhancing from 40% to almost 60% after 4h (Figure 2A). Moreover, we could reproduce the regulation of migration by *ID3* in a Boyden chamber system for A375, SKmel28 and WM266-4 cell lines, confirming the role of ID3 in this cell function (Supplementary Figure 5). Interestingly, neither *ID3* knockdown nor *ID3* overexpression had a significant impact on melanoma cell lines’ proliferation rate as quantified by alamar blue staining over a period of 5 days (Figure 2B). Similarly, none of these cell lines showed a significant difference on cell cycle states, which was analysed by flow cytometry after staining the cells with propidium iodide (Figure 2C).

In line with the migration phenotype, *ID3* knockdown cell lines presented a downregulation of mesenchymal markers’ expression (*FN1*, *SLUG* and *SNAIL*) compared to the control lines (Figure 2D). The analysis of AKT and ERK activation however did not show any difference in their phosphorylation status when *ID3* was either silenced or overexpressed (Supplementary Figure 4C), suggesting that the migration phenotype may be AKT and ERK-independent. To identify which pathways were regulated by ID3, we analysed with Ingenuity Pathway Analysis (IPA) the 118 regulated genes in WM266-4 *ID3* overexpressing cell line (compared to WM266-4 EV) (Supplementary Figure 4D). Among the top pathways, we found cyclins and cyclin dependent kinases. This corroborates the known function of ID3 in cell cycle regulation [20]. Many genes were found related to epithelial adherens junctions, gap junctions, FAK, integrins and actin signaling. These molecules are involved in either cell-cell interations or cytoskeleton organisation, and could explain the effect of ID3 regulation on cell migration. Another important regulated gene is *EIF4*, which encodes a multi-subunit protein complex facilitating the recruitment of mRNA to the ribosome. Therefore, ID3 could also be involved in protein translation mechanisms. Last, many molecules associated with general cancer mechanisms were regulated including bcatenin, HIF1α, MYC or aurora kinase A.

In the last part, we focused on the expression of resistance-associated genes *SOX10* and *MITF*. We observed a 2 to 4-fold upregulation in *ID3* knockdown cell lines compared to their control lines (Figure 2E) and a downregulation of half in the *ID3* overexpressing line WM266-4 (Figure 2F). However, a western blot on 11 melanoma cell lines showed a heterogenous basal expression pattern for these two genes and no correlation was found with either ID3 levels or with the *BRAF* or *NRAS* mutational status of these cell lines (Supplementary Figure 6A). Nevertheless, we selected the five cell lines which expressed similar levels of both SOX10 and MITF (SKmel23, SKmel28, SKmel30, C32 and MeWo) and we observed an inversed tendency between SOX10/ MITF expression and ID3 expression (Supplementary Figure 6B). For instance, SOX10 and MITF levels were low in SKmel28 and C32 but ID3 level was high. Conversely, SOX10 and MITF levels were high in SKmel23, SKmel30 and MeWo but ID3 levels were rather low. These data presented a significant inversed tendency with an r value of -0.9. Finally, the analysis of an online database of metastatic melanoma patient-derived tissues (GEO accession number: GDS3966) confirmed similar inversed tendency between SOX10 and ID3 expression on one hand and between MITF and ID3 expression on the other hand (46 samples and 48 samples respectively). These expression values differences were significant (pval = 0.0028 and pval < 0.0001 respectively) although the r values remained rather low (*r* = -0.4313 and *r* = -0.5963 respectively) (Supplementary Figure 6C).

In sum, these results show that ID3 controls melanoma cell migration and regulates mesenchymal markers, without affecting cell proliferation or cell cycle states. They also show that ID3 expression inversely correlates with that of SOX10 and MITF in melanoma cell lines and in melanoma patient samples.

## DISCUSSION

In this report, we show that melanoma cells upregulate *ID3* expression (and also *ID1* and *ID2*) in response to vemurafenib (or to the combination vemurafenib + trametinib) *in vitro* and in patients’ tumors. We also showed that modulation of *ID3* expression regulates the sensitivity of the cells to vemurafenib. Interestingly, *ID3* expression has been involved in non-small cell lung cancer resistance to chemotherapy [25]. *ID3* expression is also higher in a model of doxorubicin-resistant melanoma cell line compared to its control cell line [26].

Although the mechanisms by which vemurafenib regulates ID3 are not yet known, suppression of *SOX10* was described to activate the TGFβ-EGFR-PDGFRB signaling and to lead to BRAF inhibitor resistance [27]. Our gene expression analysis of vemurafenib-treated cells did not identify this signaling but suggested however the involvement of p53, Wnt/βcatenin signaling or even the glycolysis pathway. ID3 protein is involved in cell cycle regulation and DNA damage, therefore its upregulation under vemurafenib treatment could have an effect on these cell functions. Expression of ID proteins correlates with p53 expression in cancer cells and protein–protein interaction between p53 and ID3 was already described [28, 29]. More recently, a positive regulation of ID4 (the fourth member in the ID family) promoter by mutant p53 was proposed in breast cancer [30]. Although no direct link between Glycolysis and ID3 was described so far, Wnt/βcatenin pathway was shown to regulate ID3 in fibroblasts and myoblasts [31, 32].

It should be noted that SKmel28 cell line carries mutations on *PTEN* and *p53* in addition to *BRAF*. Mutations in these important tumor suppressor genes seem not to influence the sensitivity to vemurafenib in comparison to A375 or WM266-4 (Figure 1D). They also do not seem to influence the *ID3*-mediated regulation of cell migration (Figure 2A). Nevertheless, as discussed above, we cannot rule out the possibility of a molecular link between ID3 and PTEN or p53 pathway.

In addition, our findings present a regulation of melanoma cell migration by *ID3* without influencing their proliferation or their cell cycle states. This data is in line with a study describing different roles for *ID3* in migration and proliferation of prostate cancer cells [33]. Although we show a regulation of genes involved in cell migration after ID3 modulation, the precise ID3 binding partners or downstream targets are not clearly identified. Our data suggest an ID3-mediated regulation of many genes associated with protein translation such as EIF4. Deeper investigation on the link between ID3 and EIF4 should increase our understanding of this mechanism. Our data also suggest a repression of SOX10 by ID3 and a role of the ID3-SOX10 axis in melanoma drug resistance. ID proteins function as heterodimers with other basic HLH transcription factors, especially E-proteins such as E2A, and inhibit their transcriptional activity by preventing their binding to the DNA [34]. Based on the known interaction between ID3 and E2A in the maintenance of cell multipotency [35] and on the presence of three E-box DNA motifs in *SOX10* promoter, we hypothesize an *ID3*-mediated regulation of *SOX10* via *E2A*.

Finally, two hypotheses can be formulated to explain *ID3* upregulation after the drug treatment: i. High ID3 expressing cell sub-population already exists in the naïve tumor and will be selected by the treatment or ii. ID3 expression is upregulated in all cells by the treatment. One argument in favor of the second hypothesis is that WM266-4 cell line, which does not express ID3 upregulates it upon vemurafenib treatment. However, more investigation on a single cell level should help answering this question. For example quantification of ID3 positive cell number in patient's tumor samples before and after treatment (immunostaining) or in melanoma cell lines treated or not with vemurafenib (flow cytometry) could be performed.

Ultimately, we found that the absence of *ID3* sensitizes melanoma cells to vemurafenib treatment, suggesting that *ID3* or *ID3* downstream targets’ pharmacological inhibition may help improving current treatment targeting resistant melanoma.

## MATERIALS AND METHODS

### Cell lines and plasmids

Human melanoma cell lines (A375, C32, HT144, MeWo, SKmel28, SKmel23, SKmel30, SKmel103, SKmel147, SKmel173, WM266-4) were cultured in DMEM (Gibco, Life Technologies) with 10% FBS (Biochrom), 0.1mM β-mercapthoethanol (Gibco, Life Technologies), 1% non-essential amino acids (NEAA) and 1% Penicilin/Streptomycin (Sigma-aldrich). Normal human melanocytes (NHM) were isolated from donor foreskins according to the ethical regulation (Ethics committee II, University Medical Center Manheim, Germany) and were cultivated in medium 254 (Gibco, Life Technologies) supplemented with 100x human melanocyte growth supplement (HMGS) (Gibco, Life Technologies). Human neural crest cells were derived from hiPSC following a previously published protocol [23]. All cell lines were cultured in humidified incubator at 37°C and 5% CO2. Cell lines were sub-cultured every 3–5 days when they were 80% confluent.

A375, HT144 and SKmel28 cell lines were transduced with a lentiviral expression vector (pLKO.1) encoding for a human *ID3* shRNA or a non-targeting shRNA (Sigma-Aldrich). WM266-4 cell line was transduced with either an empty lentiviral vector (pLX304) or the vector encoding for human *ID3* (Addgene).

### Microarray gene expression profiling

Total RNA from three independent experiments was isolated from primary melanocytes, human neural crest cells, melanoma cell lines A375, SKmel28 and WM266-4 (+/- treated with vemurafenib) and A375 *ID3* knockdown/ NT cell lines, and purified with RNeasy kit (Qiagen). Labeled RNA was hybridized to whole-genome BeadChip Sentrix arrays HumanHT-12 v4 from ILLUMINA (Santa Clara, CA, USA) following the manufacturer's indications. Microarray scanning was carried out using an iScan array scanner.

As test for significance, a Bayes test was used on the bead expression values of the two groups of interest. The average expression value is calculated as mean of the measured expressions of beads together with the standard deviation of the beads. After selecting the genes, which *P*-values were inferior to 0.05, log2-expression values of the differentially expressed genes were represented.

### Gene expression datasets were uploaded on GEO database (GSE104849)

Regulated genes in vemurafenib-treated cells (compared to DMSO) or in *ID3* overexpressing cells (compared to control vector) were then uploaded to IPA software (Ingenuity Pathway Analysis) to evaluate the most regulated signaling pathways.

### Lentiviral particles tranduction

Lentiviral particles were produced in HEK293T cells. HEK293T cells were approximately 60% confluent on the day of transfection. The plasmid with the gene of interest along with packaging plasmids VSV-G and Δ8.9 was mixed in the DMEM and X-treme GENE^®^ solution. The mixture was incubated for 30 min at room temperature and then added to HEK293T producer cells. The collected supernatant was concentrated by ultracentrifugation and the virus was used to infect the cells. The virus production was done in a Biosafety level II laboratory, according the safety instruction. After 48h of transduction the cells were washed with PBS and normal fresh medium was added to the cells.

### Antibiotic selection

Cell lines with *ID3* shRNA were selected by using puromycin (0.5-0.8 μg/ml) and cell lines with *ID3* overexpressing vector were selected using blasticidin (5-8 μg/ml) for 4-6 days.

### RNA isolation and cDNA synthesis

Total RNA isolation from melanoma cell lines (A375, SKmel28, HT144 and WM266-4), neural crest cells (D1NC) and normal human melanocytes (NHM) was done using RNeasy Mini kit (Qiagen) according to the manufacturer's protocol. The RNA was treated with DNase I on the column. RNA concentration and quality were measured by NanoDrop ND1000 spectrophotometer. cDNA was synthesized using the Revert Aid First Strand cDNA synthesis kit (Thermo scientific) according to the manufacturer's protocol.

### qPCR

Quantitative real-time PCR (qPCR) was performed using SYBR Green (Applied Biosystems, Life technologies) on a 7500 real-time PCR system (Applied Biosystems, Life technologies). RNA samples were isolated from *ID3*-engineered melanoma cell lines (A375, SKmel28, HT144 and WM266-4). In all experiments, rRNA 18s was used as the housekeeping gene and the values were normalized to it. Relative gene expressions were quantified by calculating (ΔΔCt). Primers used are as follow: *18S* F: GAGGATGAGGTGGAACGTGT, *18S* R: TCTTCAGTCGCTCCAGGTCT, *ID3* F: GGA GCTTTTGCCACTGACTC, *ID3* R: TTCAGGCC ACAAGTTCACAG, *SOX10* F: GGCTTTCTGTCTGG CTCACT, *SOX10* R: TAGAGGGTCATTCCTGGGGG, *MITF* F: GCTCACAGCGTGTATTTTTCC, *MITF* R: TC TCTTTGGCCAGTGCTCTT, *FN1* F: GGTGAC ACTTATGAGCGTCCTAAA, *FN1* R: AACATGTA ACCACCAGTCTCATGTG, *SLUG* F: TGGTCAA GAAACATTTCAACGCC, *SLUG* R: GGTGAGGATC TCTGGTTTTGGTA, *SNAIL* F: GAGGCGGTGGC AGACTAG, *SNAIL* R: GACACATCGGTCAGACCAG.

### Western blot

Protein samples were extracted from human melanoma cell lines (A375, C32, HT144, MeWo, SKmel28, SKmel23, SKmel30, SKmel103, SKmel147, SKmel173, WM266-4) and from *ID3*-engineered melanoma cell lines (A375, SKmel28, HT144 and WM266-4) by using 1% Triton-X-100 lysis buffer containing a protease inhibitor cocktail (complete mini, Roche). The protein concentration was determined using the Pierce BCA protein assay kit (Thermo scientific). Proteins were resolved on SDS-PAGE and transferred to PVDF membranes (Merck Millipore). Later the membrane was probed with primary antibodies and then with HRP conjugated secondary antibody. The bands were visualized using Luminata Forte western HRP substrate (Merck Millipore) according to manufacturer's protocol. The band intensities were quantified using ImageJ software (Fiji). The primary antibodies used are as follow: ID3 (Calbiochem), SOX10 (Abcam), MITF (Abcam), GAPDH (CST).

### Migration assay

Cell migration was studied on all *ID3*-ingeneered melanoma cell lines (A375, SKmel28, HT144 and WM266-4) via two methods. Culture silicone inserts from Ibidi (scratch-like assay), 70 000 cells were seeded for 24 h and were serum-starved overnight. The inserts were removed and cell migration was observed every 4 hours up to 28 hours. TScratch software was used for the quantitative analysis of the data. Alternatively, Boyden chamber system was used according to manufacturer's protocol (Trevigen).

### Cell proliferation and cell cycle analysis

Cell proliferation was measured in all *ID3*-engineered melanoma cell lines (A375, SKmel28, HT144 and WM266-4) using Alamar blue (Invitrogen) for up to 6 days. 2500 or 5000 cells were seeded in triplicates in a 96-well plate. After 24 hours Alamar blue (10% of the medium) was added and the plates were incubated for 4 hours at 37°C. Fluorescence was measured with excitation wavelength at 530-560 nm and emission wavelength at 590 nm using the Tecan Infinite 200 Pro plate reader. For cell cycle analysis, A375, SKmel28, and WM266-4 *ID3*-engineered cells were seeded (4–6 × 10^5^) in 6-well plates in triplicates and incubated at 37°C for 18-24 hours. After cell collection and centrifugation, cell pellets were resuspended in ice-cold PBS and fixed with pre-cooled 70% ethanol. Cells were then washed with ice-cold PBS and treated with RNase for 30 min at 37°C. Propidium iodide (50 μg/ml) was used to stain the cellular DNA. Cell cycle stages were analysed by flow cytometry (Canto, Becton-Dickson).

### Cell viability

Human melanoma cell lines (A375, HT144, SKmel28, and WM266-4) and *ID3*-engineered cell lines (A375, SKmel28, HT144 and WM266-4) were seeded in 96-well plates (2500 cells per well). After 16-18 hours, increasing concentrations (0.001-10 μM) of Vemurafenib (PLX4032) or DMSO were added to the cells. Combination treatement was performed with Vemurafenib and Trametinib (GSK1120212) at ratio 1:1. Cell viability was measured every 24 hours up to 4 days using Alamar blue as described above.

## SUPPLEMENTARY MATERIALS FIGURES AND TABLES


