# Supplementary material for: New role of ID3 in melanoma adaptive drug-resistance
Source: Oncotarget. 2017 Nov 27;8(66):110166–75. doi: 10.18632/oncotarget.22698 (PMC5746373; doi:10.18632/oncotarget.22698)
Supplement: Supplementary file 1 [file oncotarget-08-110166-s001.pdf]

New role of ID3 in melanoma adaptive drug-resistance

SUPPLEMENTARY MATERIALS

A

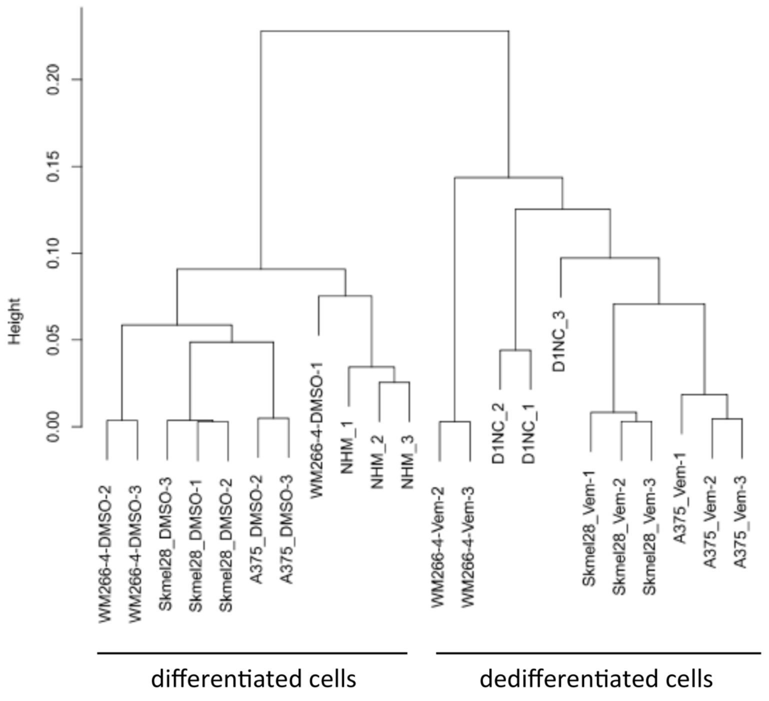

B

	Ingenuity Canonical Pathways	-log(p-value)
cell cycle	Cell Cycle Control of Chromosomal Replication	1.47E+01
	Role of CHK Proteins in Cell Cycle Checkpoint Control	6.61E+00
	G2/M DNA Damage Checkpoint Regulation	6.04E+00
	G1/S Checkpoint Regulation	3.39E+00
	Cyclins and Cell Cycle Regulation	6.11E+00
DDR	Mismatch Repair in Eukaryotes	6.18E+00
	ATM Signaling	5.99E+00
	DNA damage-induced 14-3-3σ Signaling	3.19E+00
	DNA Double-Strand Break Repair by Homologous Recombination	1.45E+00
	p53 Signaling	3.83E+00
	Glycolysis	1.84E+00
	Wnt/βcatenin Signaling	1.51E+00

Supplementary Figure 1: Vemurafenib treatment induces a dedifferentiation profile. (**A**) Unsupervised hierarchical clustering of the following samples: neural crest cells (D1NC), normal human melanocytes (NHM) and melanoma cell lines (A375, SKmel28, WM266-4) treated with 3 µM vemurafenib for 72 h (Vem) or with DMSO (DMSO). Dendrogram was performed using Pearson correlation and average linkage method. Cluster including NHM was labeled "differentiated cells" and cluster including D1NC was labeled "dedifferentiated cells". (**B**) Ingenuity Pathway Analysis (IPA) performed on 431 regulated genes in vemurafenib-treated melanoma cell lines compared to DMSO.

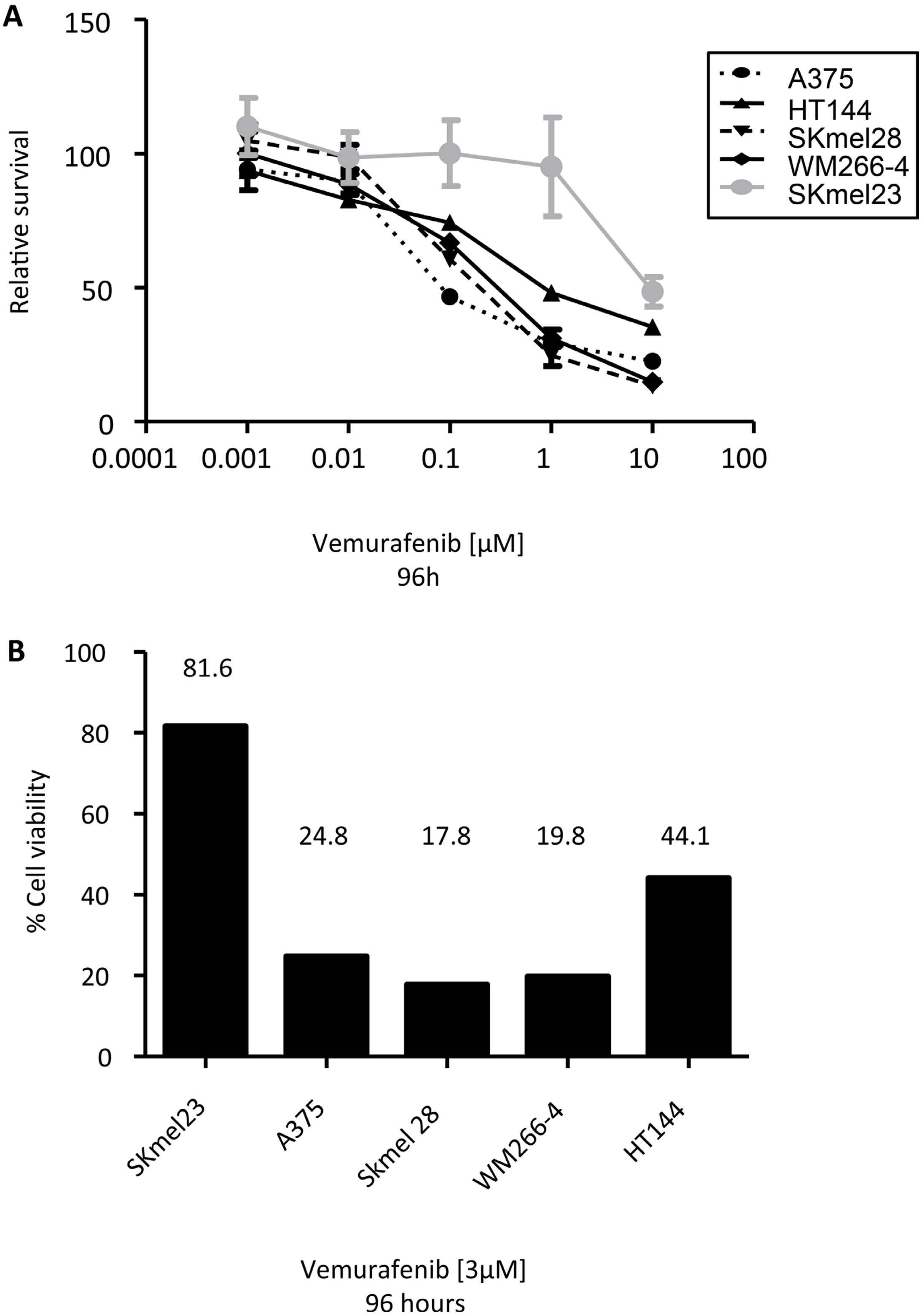

Supplementary Figure 2: Cell viability assay on melanoma cell lines after 96 h vemurafenib treatment. (**A**) A375, SKmel28, WM266-4, HT144 and SKmel23 cell lines were treated with vemurafenib (0.001 to 10 µM) for 96 h. Cell viability was assessed by Alamar blue staining. Relative survival represents the ratio of cell viability of treated cell lines over the cell lines treated with DMSO. (**B**) Graph represents the percentage of cell viability after 96 h treatment with 3 µM vemurafenib. Data represent mean ± SD of biological triplicates.

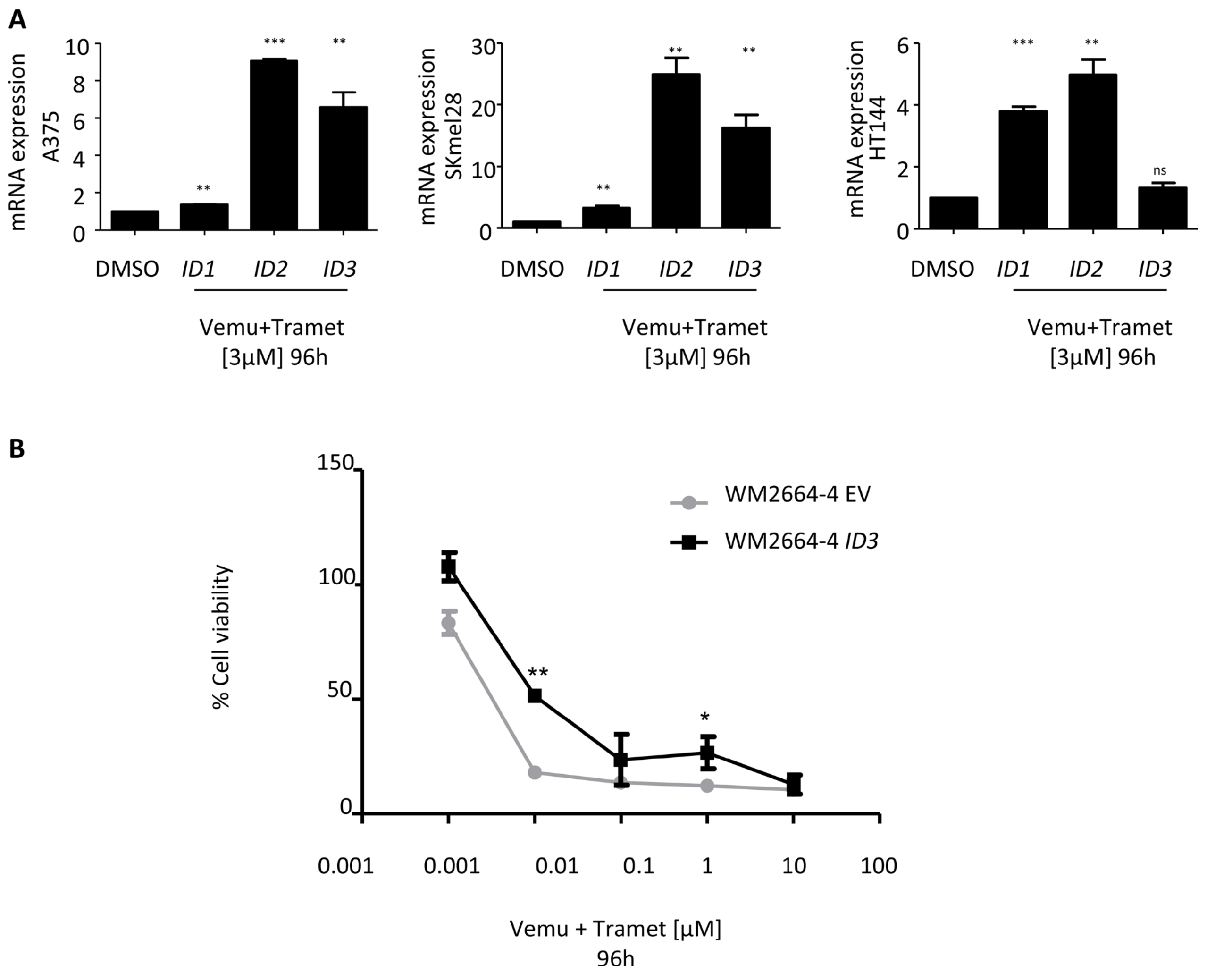

Supplementary Figure 3: Effect of combination treatment vemurafenib and trametinib. (**A**) *ID1, ID2*, and *ID3* mRNA expression was analysed by qPCR in human melanoma cell lines (A375, SKmel28, and HT144) treated with vemurafenib (3 µM) plus trametinib (3 µM) for 96 h. rRNA *18S* was used as an endogenous expression control and DMSO treated cells were used as reference sample. (**B**) Graph represents the effect of combination treatment (0.001-10 µM) after 96 hours on the viability of *ID3* overexpressing cell line (WM266-4 *ID3*) or cell line transduced with non targeting shRNA (WM266-4 NT), assessed by Alamar blue staining. Data represent mean ± SD of biological triplicates. $^{*}P < 0.05$, $^{**}P < 0.01$, $^{***}P < 0.001$.

A

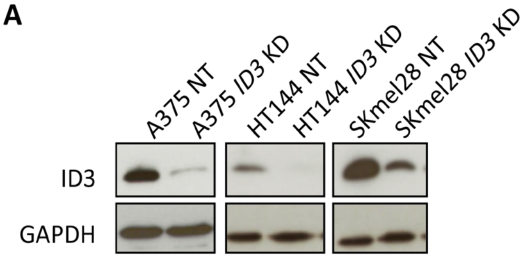

B

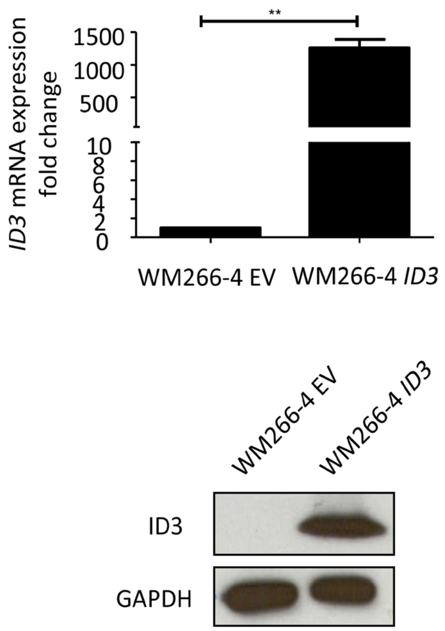

C

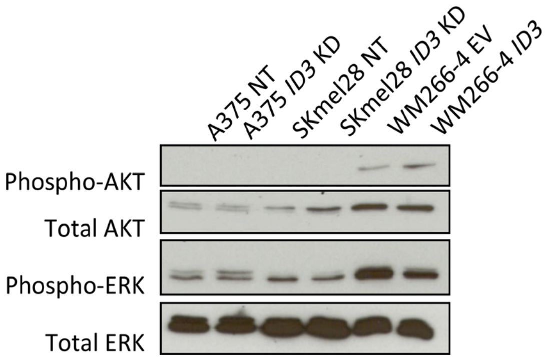

D

Ingenuity Canonical Pathways	-log(p-value)	Molecules
Cyclins and Cell Cycle Regulation	3.97	CCNA2,PA2G4,CCNB2,CDK1,CDK2,E2F2,CCNB1,CDC25A,SKP2
Remodeling of Epithelial Adherens Junctions	4.51	NME1,ARPC1B,ACTA2,TUBB4B,ACTB,CTNNA1,TUBA1C,CTNNB1,ACTG1
FAK Signaling	2.56	ITGB1,TLN2,ACTA2,RRAS,ASAP1,ACTB,HMMR,ACTG1
Integrin Signaling	1.81	ITGB1,TSPAN3,TLN2,ARPC1B,TSPAN7,ACTA2,RRAS,ASAP1,ACTB,ACTG1,ITGB3
Actin Cytoskeleton Signaling	1.34	ITGB1,DIAPH1,TLN2,ARPC1B,ACTA2,RRAS,ACTB,BRK1,ACTG1,GNG12
Gap Junction Signaling	1.28	ACTA2,TUBB4B,RRAS,ACTB,ADCY1,TUBA1C,CTNNB1,ACTG1
Molecular Mechanisms of Cancer	1.69	ITGB1,PA2G4,RRAS,GNA11,CTNNA1,HIF1A,AURKA,CDC25B,MYC,TGFBR2,FANCD2,ADCY1,CTNNB1,CDK2,E2F2,CDC25A
EIF2 Signaling	4.72	RRAS,ATF5,ACTB,RPL17,RPS10,RPL26,EIF4G1,RPS29,RPL10A,MYC,ACTA2,RPS13,EIF4A1,RPL21,RPS27A,RPS2,RPLP0

Supplementary Figure 4: Validation of *ID3* knockdown and overexpression. (**A**) *ID3* expression in ID3 knockdown cell lines (A375, SKmel28 and HT144) using western blot. GAPDH was used as loading control. *ID3* was detected at 13 kDa and GAPDH at 37 kDa. (**B**) *ID3* overexpression in WM266-4 was validated using qPCR (top panel) and by western blot (bottom panel). The qPCR results are mean ± SD of biological triplicates. $^{**}P < 0.01$.(**C**) Western blot analysis of phospho-ERK and phospho-AKT (and respective total ERK and total AKT) status in *ID3* engineered cell lines. (**D**) Transcriptome analysis with Ingenuity Pathway Analysis (IPA) of the 118 regulated genes in ID3 overexpressing WM266-4 melanoma cell line compared to the control line expressing a non-targeting shRNA.

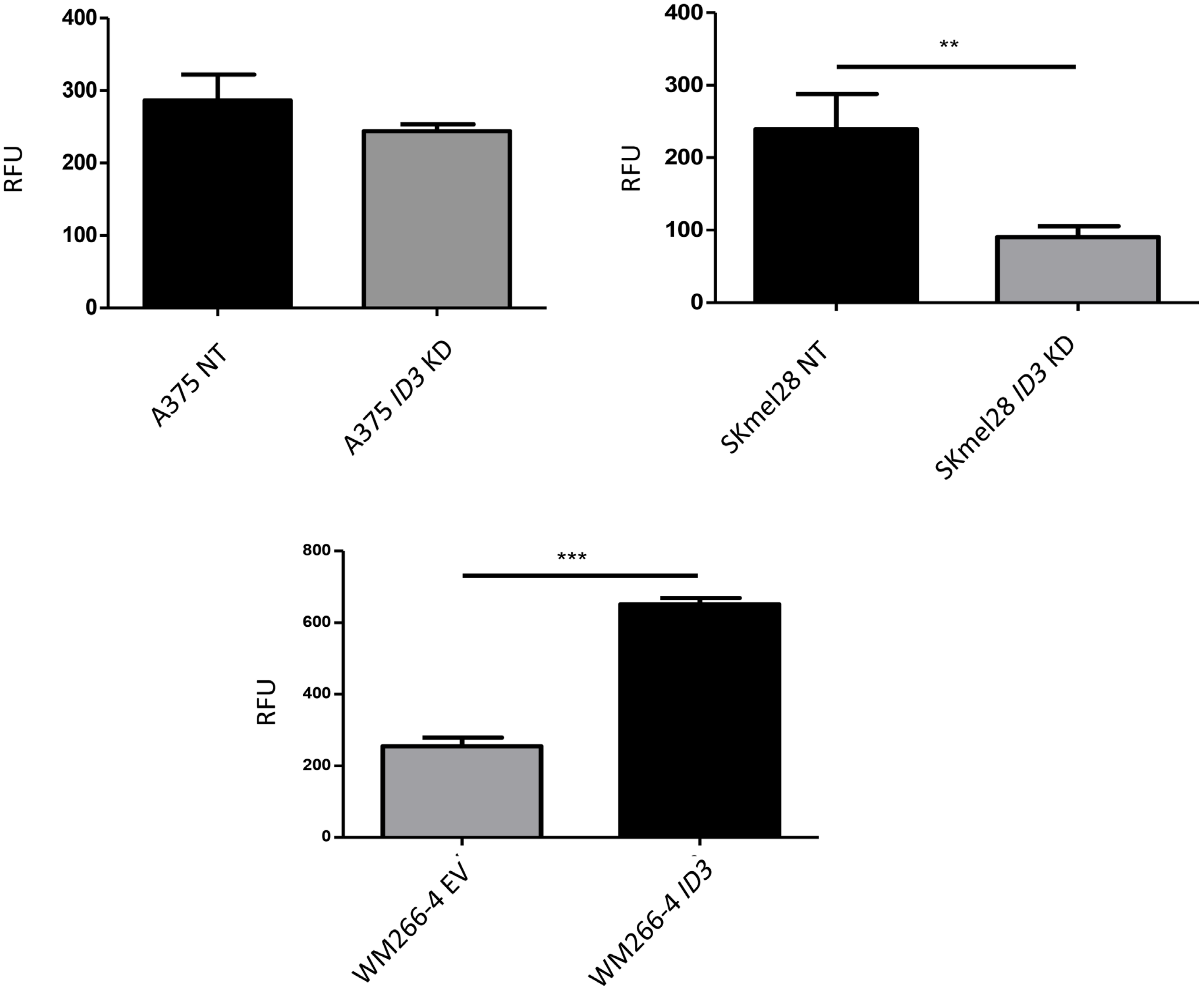

Supplementary Figure 5: Effect of *ID3* knockdown or overexpression on cell migration. Migration analysis using Boyden chambers of *ID3* engineered cell lines (A374, SKmel28 and WM266-4). The graph represents the mean ± SD of biological triplicates. $^{*}P < 0.05$, $^{**}P < 0.01$, $^{***}P < 0.001$.

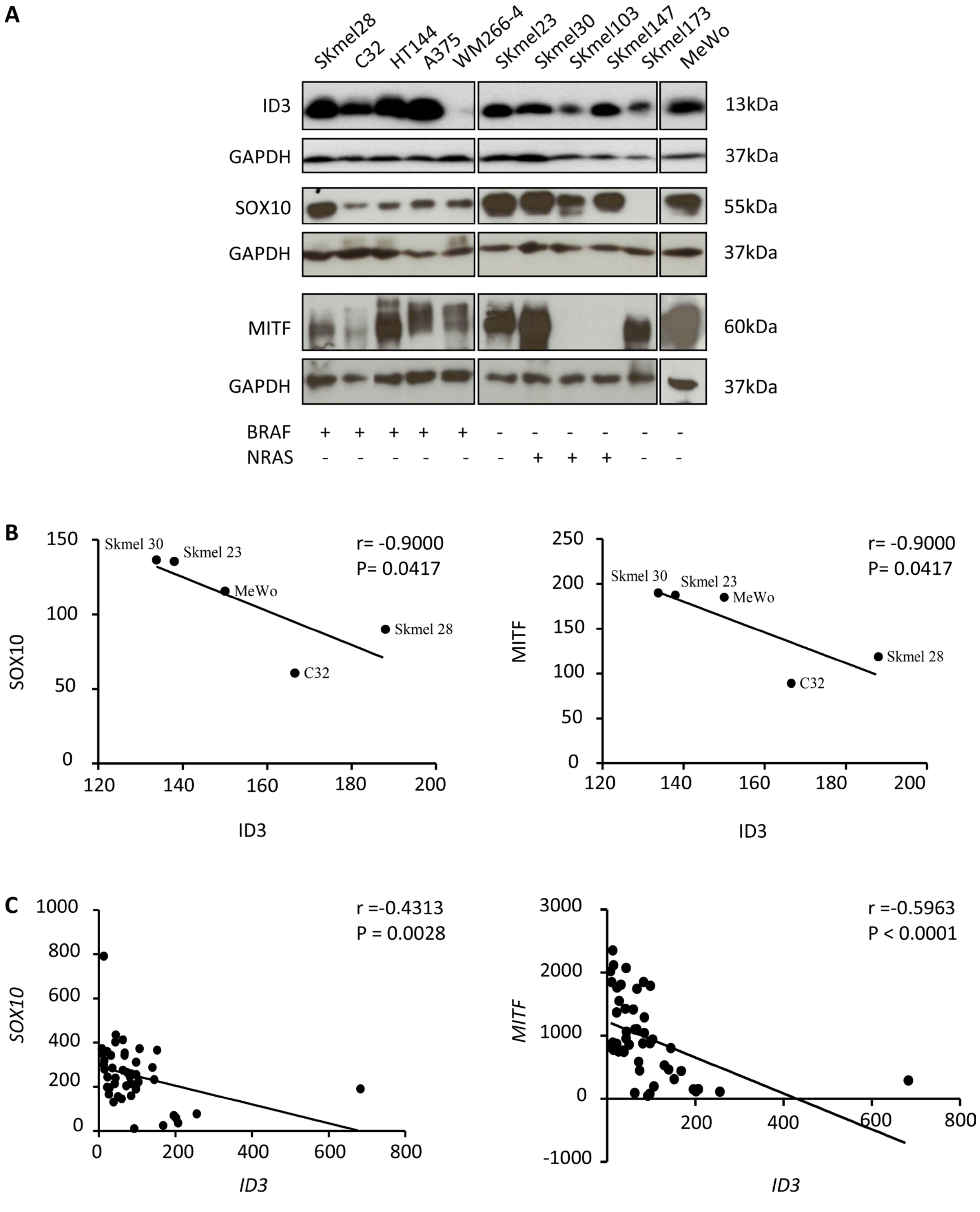

Supplementary Figure 6: Protein expression of ID3, MITF and SOX10 in melanoma samples. (**A**) Protein expression of ID3, MITF and SOX10 in a panel of 11 melanoma cell lines. GAPDH was used as a housekeeping gene. Mutational status of *BRAF* and *NRAS* is reported. (**B**) Graphs represent protein expression values estimated with ImageJ software from the above western blot in a panel of 5 melanoma cell lines (SKmel23, SKmel30, SKmel28, MeWo, C32). ID3 expression was compared to either SOX10 or MITF expression. Spearman correlation was calculated and *p* value was statistically significant in both graphs. (**C**) mRNA expression values from metastatic melanoma patients were downloaded from GEO database and Spearman correlation between *ID3* and either *SOX10* or *MITF* was calculated. *P* value showed statistical significance (GEO accession number: GDS3966).

Supplementary Table 1: List of IC^{50} for all cell lines

	Vemurafenib IC50 (µM)
A375	0.1
SKmel28	0.2
WM266-4	0.4
HT144	0.9
SKmel23	9.8

A375 NT	10.1
A375 *ID3* KD	0.5
SKmel28 NT	10.3
SKmel28 *ID3* KD	1.0
HT144 NT	9.8
HT144 *ID3* KD	5.0
WM266-4 EV	0.3
WM266-4 *ID3*	0.7
